# Binding selectivity of dibenzo-18-crown-6 for alkali metal cations in aqueous solution: A density functional theory study using a continuum solvation model

**DOI:** 10.1186/1752-153X-6-84

**Published:** 2012-08-08

**Authors:** Chang Min Choi, Jiyoung Heo, Nam Joon Kim

**Affiliations:** 1 Department of Chemistry, Chungbuk National University, Chungbuk, 361-763, South Korea; 2 Department of Biomedical Technology, Sangmyung University, Chungnam, 330-720, South Korea

**Keywords:** Density functional theory, Crown ether, Continuum solvation model, Binding selectivity

## Abstract

**Background:**

Dibenzo-18-crown-6 (DB18C6) exhibits the binding selectivity for alkali metal cations in solution phase. In this study, we investigate the main forces that determine the binding selectivity of DB18C6 for the metal cations in aqueous solution using the density functional theory (DFT) and the conductor-like polarizable continuum model (CPCM).

**Results:**

The bond dissociation free energies (BDFE) of DB18C6 complexes with alkali metal cations (M^+^-DB18C6, M = Li, Na, K, Rb, and Cs) in aqueous solution are calculated at the B3LYP/6-311++G(d,p)//B3LYP/6-31 + G(d) level using the CPCM. It is found that the theoretical BDFE is the largest for K^+^-DB18C6 and decreases as the size of the metal cation gets larger or smaller than that of K^+^, which agrees well with previous experimental results.

**Conclusion:**

The solvation energy of M^+^-DB18C6 in aqueous solution plays a key role in determining the binding selectivity of DB18C6. In particular, the non-electrostatic dispersion interaction between the solute and solvent, which depends strongly on the complex structure, is largely responsible for the different solvation energies of M^+^-DB18C6. This study shows that the implicit solvation model like the CPCM works reasonably well in predicting the binding selectivity of DB18C6 in aqueous solution.

## Introduction

Crown ether is one of the most well-known host molecules, which exhibits the binding selectivity for alkali metal and alkali earth metal cations in solution phase [1,2]. In particular, 18-crown-6 (18C6) and DB18C6, which are the first crown ethers synthesized by Pedersen in 1967 [3], have the strongest binding affinity to a potassium cation (K^+^) among alkali metal cations. With the similarity of the sizes between K^+^ and the cavity of crown ether, it had long been believed that the binding selectivity comes from the size relationship between the metal cation and the cavity. However, it was reported that under identical experimental conditions K^+^ bound strongly to all crown ethers (12-crown-4 to 24-crown-8) irrespective of the ring size among other cations such as Na^+^, Ca^2+^, and NH_4_^+^[4]. Moreover, it turned out from quantum theoretical and gas-phase experimental studies that the intrinsic binding affinity of 18C6 or DB18C6 in the gas phase is stronger for smaller metal cations such as Li^+^ or Na^+^ rather than K^+^[5-8]. This discrepancy between the solution- and gas-phase results indicates that the solvation effects strongly influence the binding selectivity of crown ethers in solution phase [9].

To understand the role of solvation and thereby the origin of the binding selectivity of crown ethers in solution phase, many experimental and theoretical studies have been performed.[9-13] A combined molecular mechanics and dynamics study on the cation selectivity of DB18C6 in methanol was carried out [12]. An *ab* initio quantum mechanical calculation was also performed to estimate the reaction enthalpies of the cation exchange reactions where alkali metal cations and the crown ether-metal cation complexes were hydrated by up to four water molecules [9,13]. The authors insisted that the cation selectivity of 18C6 in aqueous solution is the result of a delicate balance of the forces that the cation experiences while the crown ether and solvent molecules compete for the cation in solution. The solvation of M^+^-18C6 itself was reported to weakly influence the energetics of the exchange reaction [9].

However, by re-examining the energetics of the exchange reaction using the experimental information, Armentrout and coworkers [5] pointed out that the different extent of stabilization of M^+^-18C6 complexes by solvation should be considered to determine the true aqueous selectivity. Rizzo and coworkers [11] also proposed that the solvation energy of M^+^-DB18C6 relative to that of bare metal cation, which depends strongly on the complex structure, primarily controls the ion selectivity of crown ethers. However, the numbers of solvent molecules used in those studies were too small, possibly due to the high cost of calculation using the explicit solvation model, to fully understand the solvation effects.

As an alternative approach in describing the solvation effects at an *ab* initio level, the continuum solvation model has drawn much attention due to its flexibility and efficiency [14,15]. Compared to the explicit solvation model arranging a few solvent molecules around the solute, the continuum model places a solute molecule in a solvent cavity surrounded by a polarizable continuum, whose reaction field modifies the energy and properties of the solute. Hence, the calculations using the continuum solvation model are cheaper, simpler, and more convenient than those using the explicit solvation model.

Some popular approaches of the continuum model include the apparent surface charge (ASC) method, the multipole expansion methods, the generalized Born approximation, the image charge methods, and the finite element and finite difference methods [15]. The polarizable continuum model (PCM) is a prototype of the ASC approach, where the electrostatic interactions with the continuum are modeled by a charge density, σ, at the surface of a solvent cavity. In this PCM family, there are the original PCM (D-PCM), the integral equation formalism PCM (IEP-PCM), the surface and volume polarization for electrostatics (SVPE), the surface and simulation for volume polarization for electrostatics (SS(V)PE), and the conductor-like screening model (COSMO), all of which differ from each other in the electrostatic expressions describing the ASC density [15,16].

One of the most successful continuum models in terms of accuracy and applicability is the CPCM model based on the COSMO [17]. Our choice of the CPCM in this study is due to the followings: First, the CPCM provides energy gradients, allowing geometry optimizations in solution. Moreover, its implementation in the Gaussian package makes it possible to perform Hartree-Fock (HF) and density functional (DF) energy calculations and geometry optimizations with the molecular wave functions provided by Gaussian packages for isolated systems [18]. Second, the CPCM provides the solvation energy as a sum of non-electrostatic and electrostatic interaction energies. The non-electrostatic energy is also given as a sum of cavitation, dispersion, and repulsion energies. Those turn out to be quite useful in this study to analyze the relative importance of those interaction energies in determining the solvation energy. Third, it was reported that the hydration free energies of neutral and charged molecules calculated using the CPCM model agreed well with the experimental data [19-22]. In particular, the CPCM model has been successfully used for crown ether complexes with metal cations [23]. The conformational analysis of 18-azacrown-6 complexes with the late first transition series divalent metal ions in aqueous solution was carried out using the PCM model [24]. The K^+^ selectivity of the [2.2.2]-cryptand in solution was also well predicted by the CPCM model [25]. The complex binding energies of [AnO_2_(18-crown-6)]^n+^, where An = U, Np, Pu and n = 1, 2, in aqueous solution were estimated using the CPCM and COSMO model [26].

Despite those advantages and extensive uses of the continuum solvation model, only a few studies have been performed to understand the binding selectivity of crown ethers in solution phase using the continuum model [25,27,28]. Here, we investigate the binding selectivity of DB18C6 for alkali metal cations in aqueous solution by calculating the BDFEs of M^+^-DB18C6 complexes using DFT and the CPCM. DB18C6 is chosen because it has a rigid structure and exists in a small number of conformational isomers, reducing the expense of the calculation [29]. Moreover, DB18C6 complexes with alkali metal cations have recently been investigated in the gas phase using various laser spectroscopic methods [30,31]. Comparing the theoretical binding selectivity of DB18C6 with the experimental one, we verify the feasibility of calculations using the continuum solvation model in predicting the binding selectivity of crown ethers. On the basis of the agreements between the theory and experiment, we also determine the main forces that influence the aqueous binding selectivity of DB18C6.

## Results and discussion

**Figure 1:**
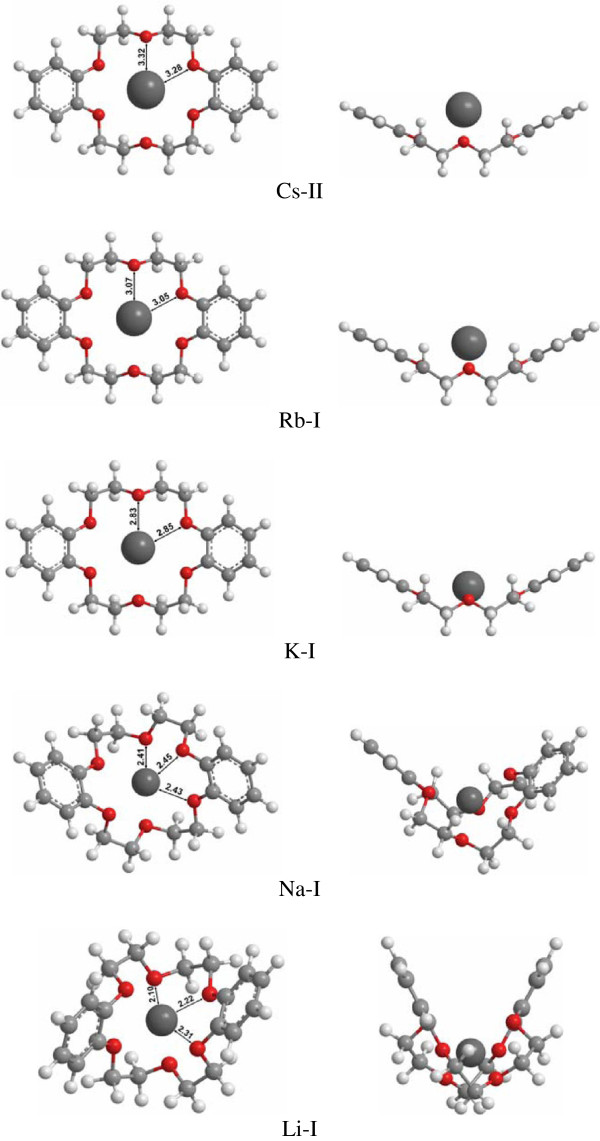
**The lowest-energy structures of M**^**+**^**-DB18C6 in aqueous solution.**

Figure1 shows the lowest energy structures of M^+^-DB18C6 in aqueous solution optimized at the B3LYP/6-31++G(d,p)//B3LYP/6-31 + G(d) level. The relative energies and Gibbs free energies for the low energy conformers in the gas and solution phases within 2 kcal/mol are listed in Table1. It is found that the lowest energy structures of M^+^-DB18C6 in aqueous solution are very similar to those in the gas phase (Additional file 1: Figure S1 in the Supporting Information). This similarity between the gas- and solution-phase structures of M^+^-DB18C6 is supported by the fact that the structural change of M^+^-DB18C6 by attachment of one water molecule is very little so that M^+^-DB18C6(H_2_O)_1_ complexes retain the gas-phase structures of M^+^-DB18C6 [11]. This is also consistent with the fact that the structures of M^+^-18C6(H_2_O)_n_ (n = 1-4) are not so different from those of M^+^-18C6 [10,32,33]. As a reason for those similarities, Lisy and coworkers [10] insisted that the 18C6…M^+^ interaction is so resilient and is still dominant with a few waters present.

**Table 1:** **Relative energies and Gibbs free energies of low energy conformers of M**^**+**^**-DB18C6 in the gas phase and aqueous solution**^** *a* **^

		**gas**			**aqueous solution**^** *b* **^	
		**Δ *E* **	**Δ *G* **		**Δ *G* **	**Δ *G* **_**s**_^** *c* **^
Li	I	0.00	0.00	I	0.00	−40.05
	II	0.16	0.67	II	0.04	−40.68
	III	0.60	0.77			
Na	I	0.00	0.00	I	0.00	−41.63
	II	1.05	0.95	IV	0.69	−41.87
	III	1.15	1.14	V	0.69	−41.90
	IV	1.39	0.93			
	V	1.40	0.97			
K	I	0.00	0.00	I	0.00	−43.87
Rb	I	0.00	0.00	I	0.00	−40.86
	II	0.50	0.34			
Cs	I	0.00	0.00	II	0.00	−38.62
	II	0.17	0.15			
	III	1.92	1.40			

^*a*^ Units in kcal/mol. ^*b*^Gibbs free energies calculated with the CPCM model. ^*c*^Gibbs free energies of solvation.

The only exception is Cs^+^-DB18C6. Although the C_s_ conformer (Cs-I) is the lowest energy conformer in the gas phase, the C_2v_ conformer (Cs-II) becomes the most stable conformer in aqueous solution. The more stabilization of the C_2v_ conformer in aqueous solution than that of the C_s_ conformer is also found in the case of Rb^+^-DB18C6. Although the C_2v_ conformer (Rb-I) is the most stable in the gas phase and aqueous solution, the energy gap between the C_2v_ and the C_s_ conformer (Rb-II) becomes larger (> 2 kcal/mol) in aqueous solution than that in the gas phase (0.34 kcal/mol).

The average distances from the metal cation to the six oxygen atoms (M-O) in K^+^-, Rb^+^-, and Cs^+^-DB18C6 increase respectively from 2.81 Å, 3.00 Å, and 3.24 Å in the gas phase to 2.84 Å, 3.06 Å, and 3.29 Å in aqueous solution. Those increases in the M-O distance may indicate the weakening of the bonds between the metal cation and the oxygen atoms in aqueous solution due to the interaction of the metal cation with the dielectric reaction field of water solvent. However, the average M-O distances of Li^+^- and Na^+^-DB18C6 in aqueous solution are nearly the same with those in the gas phase, which seems because the folded DB18C6 backbones hinder the interactions of the metal cations with the water solvent.

**Figure 2:**
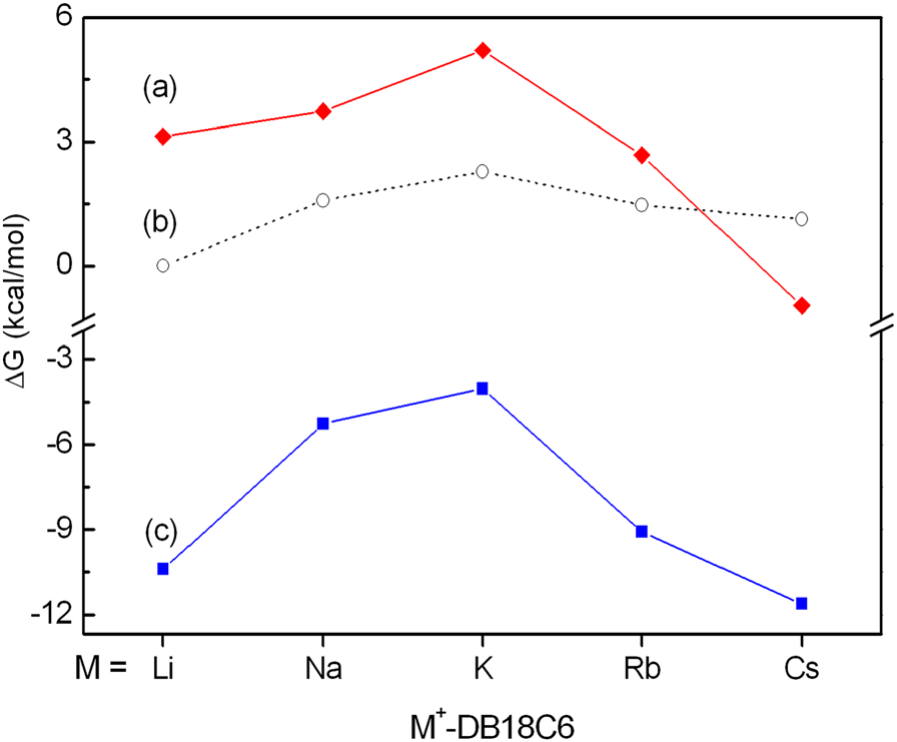
**The BDFE of M**^**+**^**-DB18C6 in aqueous solution.** (**a**) and (**c**) represent theoretical values calculated using the CPCM and MoCPCM models, respectively, and (**b**) shows the experimental values from ref. 1.

The bond dissociation energy (BDE) and BDFE of the metal cation from M^+^-DB18C6 are listed in Table2. In the gas phase the BDFE decreases as the size of the metal cation increases from Li^+^ to Cs^+^, consistent with the previous results [8]. In aqueous solution, however, the BDFE is calculated to be the largest for K^+^-DB18C6 and decreases as the metal cation in M^+^-DB18C6 gets larger or smaller than K^+^. These also agree well with the previous experimental results [34-36]. Figure2 shows the theoretical and experimental BDFEs of M^+^-DB18C6 in aqueous solution. The theoretical BDFEs qualitatively reproduce the experimental results. However, the values of Li^+^- and Cs^+^-DB18C6 exhibit relatively large discrepancies between the theory and experiment. Experimentally, Li^+^-DB18C6 has the lowest BDFE next to Cs^+^-DB18C6 but theoretically has the third largest BDFE next to Na^+^-DB18C6. Moreover, Cs^+^-DB18C6 is predicted to have the negative BDFE.

**Table 2:** **Energies and Gibbs free energies for the dissociation reaction of M**^**+**^**-DB18C6 in the gas phase and aqueous solution**^** *a* **^

		**gas**			**aqueous solution**	
		**Δ *E* **	**Δ *G* **		**Δ *G* **	**Δ *G* **^** *b* **^
Li	I	97.72	89.58	I	3.13	0.00
	II	97.03	88.38	II	2.62	
	III	96.69	88.37			
Na	I	77.56	68.78	I	3.73	1.58
	II	76.98	68.28	IV	3.49	
	III	77.00	68.23	V	3.41	
	IV	76.85	68.53			
	V	76.96	68.60			
K	I	57.98	49.06	I	5.20	2.28
Rb	I	48.74	39.85	I	2.68	1.47
	II	48.21	39.47			
Cs	I	40.22	31.53	II	−0.96	1.13
	II	40.12	31.46			
	III	38.33	30.16			

^*a*^Units in kcal/mol. ^*b*^Experimental values from ref. 1.

We assume that those discrepancies arise from an error of the CPCM model in estimating the hydration energy for metal cations. It has been reported that continuum solvation models are often inadequate for dealing with ionic solutes that have concentrated charge densities with strong local solute-solvent interactions [37,38]. In other words, although the long-range electrostatic interactions between an ionic solute and solvent are well described in the dielectric continuum model such as CPCM, specific short-range interactions between the ionic solute and the solvent molecules in the first solvation shell are not well treated in the continuum model. The specific short-range interactions of a metal cation in water arise from reorientation of the first shell water molecules. Different from water molecules in the bulk, where those are bound to each other through hydrogen bonds, the first shell water molecules surrounding the metal cation reorient themselves to bring the electronegative ends of the water dipole moments closer to the positive charge of the cation [39]. This may give rise to stronger ion-dipole interactions and hence larger hydration energy than that described in the continuum models. Indeed, we found that the hydration free energies of alkali metal cations calculated with the CPCM are smaller by 9 ~ 15 kcal/mol than the experimental values (Table S1 in Supporting Information).

One approach to handle the error in the hydration-energy calculation using the CPCM is to alter the sphere radii of solute atoms in the model so that the theoretical hydration energies may coincide well with the experimental values [40]. Here we modified the sphere radii of alkali metal cations in the united atom topological model (UAKS), which was used in our CPCM calculations. The hydration free energies of the metal cations calculated with those modified radii are listed in Additional file 1: Table S1 in Supporting Information. The BDFEs calculated with those modified radii of alkali metal cations using the CPCM model (MoCPCM) are listed in Additional file 1: Table S2 in Supporting Information. The BDFEs with the MoCPCM turn out to be all negative, possibly due to the larger increase in the hydration energy of the metal cation than that of M^+^-DB18C6 (Figure2). It is found that the BDFE of Li^+^-DB18C6 calculated using the MoCPCM is the second lowest next to that of Cs^+^-DB18C6 and the difference in their BDFE values becomes very small. Those results agree better with the experimental ones than those using the CPCM. This implies that the inaccuracy of the CPCM model in calculating the hydration energy for metal cations may indeed give rise to the discrepancies between the experimental and theoretical BDFEs of Li^+^- and Cs^+^-DB18C6.

**Figure 3:**
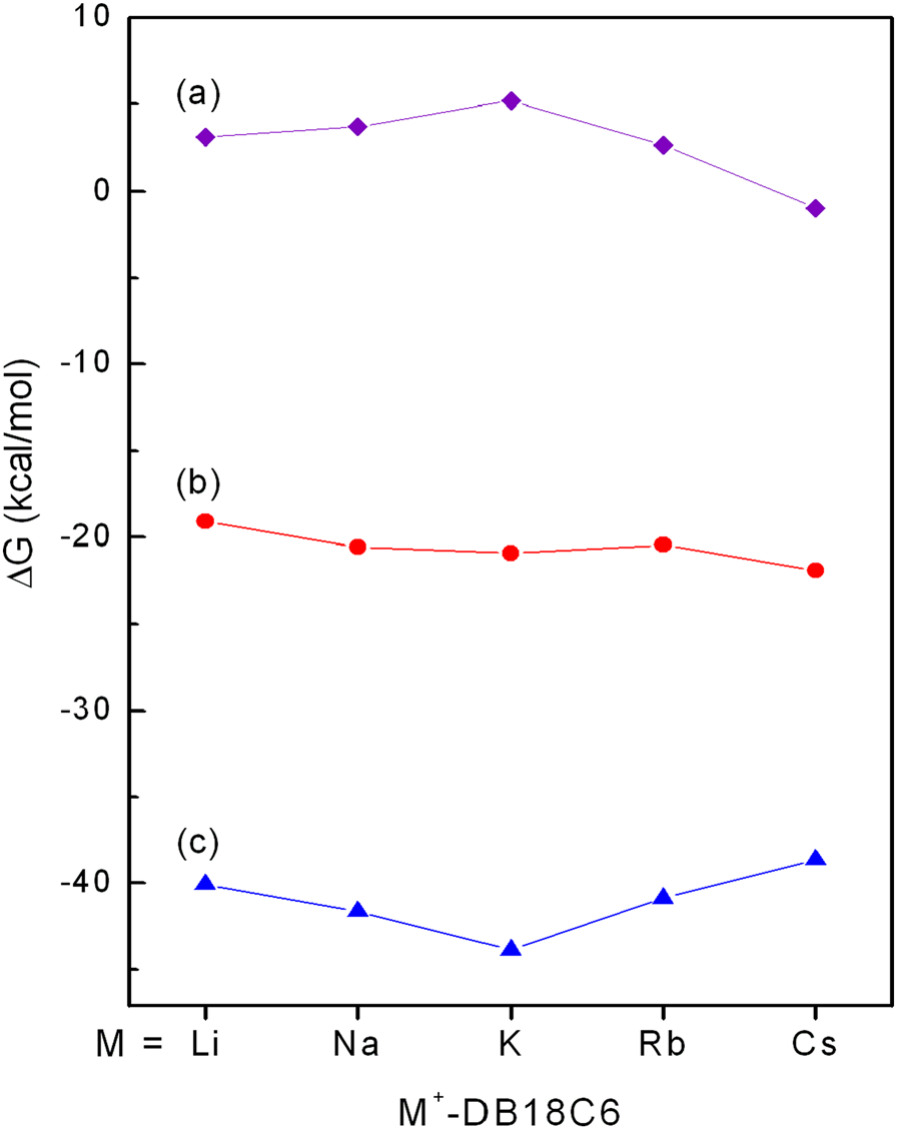
(**a) The BDFE of M**^**+**^**-DB18C6 in aqueous solution, Δ *G* **_**BD**_**( *aq* ), (b) the sum of the BDFE in the gas phase and the hydration free energy of the metal cation, Δ *G* **_**BD**_**( *g* ) + Δ *G* **_**s**_**(M**^**+**^**), and (c) the hydration free energy of M**^**+**^**-DB18C6, Δ *G* **_**s**_**(M**^**+**^**CE).**

The most interesting finding is that the strongest binding affinity of DB18C6 for K^+^ in aqueous solution is well reproduced by our theoretical calculations. Moreover, the theoretical binding affinity represents the trend of the experimental binding affinity well, which decreases as the size of the metal cation gets larger or smaller than that of K^+^[34]. On the basis of those agreements between the experiment and theory, we analyze the energy terms contributing to the BDFE to know which energy term is the most responsible for such a size effect of the metal cation on the binding affinity of DB18C6. The BDFE of M^+^-DB18C6 in aqueous solution, Δ*G*_BD_(*aq*), is given by

(1) $$\Delta G_{\mathrm{BD}}\left(aq\right)=\Delta G_{\mathrm{BD}}\left(g\right)+\Delta G_{s}\left(CE\right)+\Delta G_{s}\left(M^{+}\right)-\Delta G_{s}\left(M^{+}CE\right)$$

where Δ*G*_BD_(*g*) is the BDFE in the gas phase (Table1). Δ*G*_s_(CE), Δ*G*_s_(M^+^), and Δ*G*_s_(M^+^CE) are the solvation free energies of DB18C6, M^+^, and M^+^-DB18C6 in aqueous solution, respectively. According to the previous report [9], the binding selectivity of DB18C6 in aqueous solution results from a balance of the forces that the metal cation experiences while the crown ether and solvent molecules compete for the cation. In other words, the sum of Δ*G*_BD_(g) and Δ*G*_s_(M^+^) determines the binding selectivity, which turns out not to be true in our calculations. The sum does not exhibit the size effect of the metal cation on the binding selectivity (Figure3b), which may be due to the monotonic decrease and increase of Δ*G*_BD_(*g*) and Δ*G*_s_(M^+^) values, respectively, with increasing the size of the metal cation. Only with the addition of the solvation free energy of M^+^-DB18C6, Δ*G*_s_(M^+^CE), in Figure3c, the size effect of the metal cation emerges (Figure3a). Even the values of Δ*G*_s_(M^+^CE) themselves exhibit the size effect. Those indicate that the solvation free energy of M^+^-DB18C6 plays a critical role in determining the binding selectivity of DB18C6 in aqueous solution.

**Figure 4:**
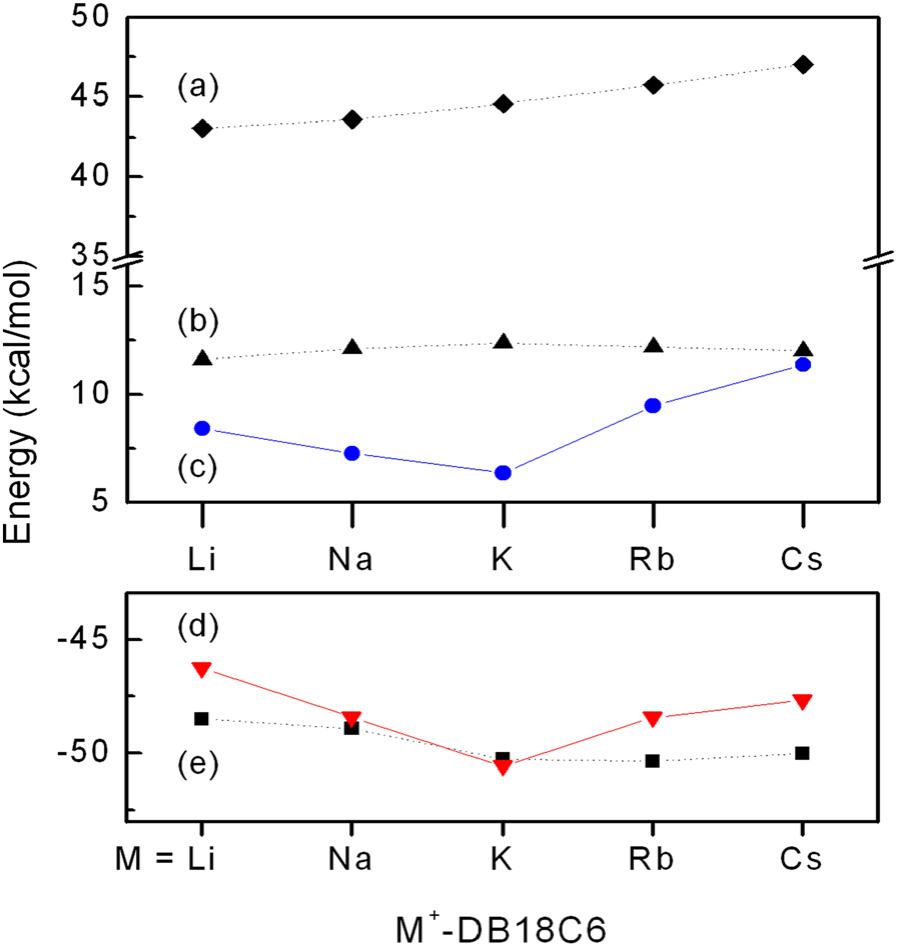
**The interaction energies contributing to the hydration free energy of M**^**+**^**-DB18C6, (a) the cavitation, (b) repulsion, (c) non-electrostatic interaction, (d) dispersion, and (e) electrostatic interaction energies.**

In the CPCM model, the solvation free energy is calculated as a sum of electrostatic and non-electrostatic interaction energies between a solute and solvent. We found that the size effect of the metal cation in the value of Δ*G*_s_(M^+^CE) arises mainly from the non-electrostatic interaction energy (Figure4c), which is a sum of the cavitation (Figure4a), repulsion (Figure4b), and dispersion energies (Figure4d). Among the three energies only the dispersion energy exhibits the size effect. The magnitude of dispersion energy is the largest for K^+^-DB18C6 and decreases as the size of the metal cation gets larger or smaller than that of K^+^.

The dispersion energy between a solute and solvent in the CPCM is calculated using a surface integral approach developed by Floris and Tomasi [41]. Thus, the dispersion energy depends on (1) the type of solute atoms, (2) the size of the solvent accessible surface area, and (3) the distances between the solute atoms and solvent. Considering that all of the solute atoms in M^+^-DB18C6s except the metal cation are the same and that a larger alkali metal cation has the larger dispersion interaction energy, we expect that the dispersion energy between M^+^-DB18C6 and solvent will increase with increasing the size of the metal cation. Moreover, the solvent accessible area of M^+^-DB18C6, which may be proportional to the cavitation energy, also increases with increasing the size of the metal cation. Thus, the numbers (1) and (2) may explain the increase in the dispersion energy from Li^+^- to K^+^-DB18C6 but not the decrease from K^+^- to Cs^+^-DB18C6. Therefore, we suggest that the dispersion-energy decrease from K^+^- to Cs^+^-DB18C6 is largely due to the number (3), the increase of the distances between the solute atoms and solvent.

**Figure 5:**
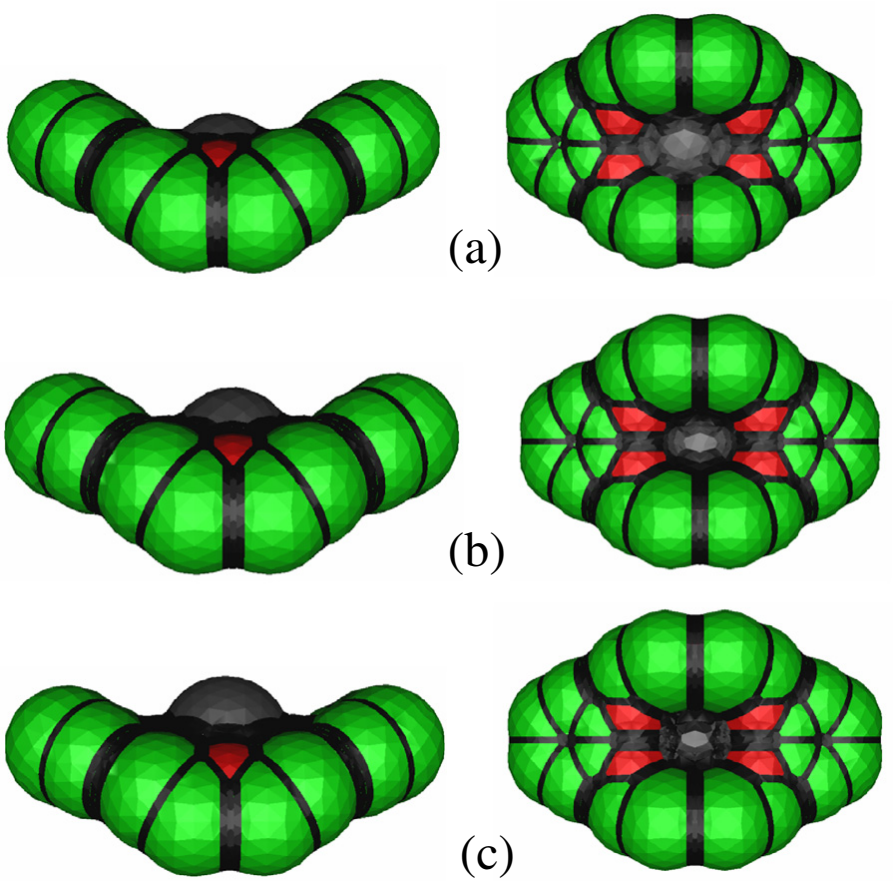
**Representations of the solvent cavities of (a) K-I, (b) Rb-I, and (c) Cs-II in aqueous solution constructed using the CPCM model and GeomView program.** The side and bottom views are shown in the left- and right-handed sides, respectively.

Although the most stable structures of K^+^-, Rb^+^-, and Cs^+^-DB18C6 in aqueous solution are the same, the boat-shaped structure with the C_2v_ symmetry, the positions of the metal cation in the cavity of DB18C6 are all different. Whereas K^+^ ion fits well inside the cavity, Rb^+^ and Cs^+^ are positioned a little above the cavity plane due to their larger sizes than the cavity (Figure1). If a metal cation is inside the cavity, the solvent above and below the cavity will have nearly the same distance to the metal cation or to the atoms of DB18C6 backbone. However, if a metal cation is located a little above the cavity, the solvent below the cavity will have longer distance to the metal cation than the solvent above the cavity. This is also true for the DB18C6 backbone atoms. The solvent above the cavity will have longer distances to the atoms of DB18C6 than the solvent below it. Those increases in the distances between the solute and solvent are better represented in Figure5, where the solvent cavities of K^+^-, Rb^+^-, and Cs^+^-DB18C6 in aqueous solution are visualized using the GeomView program [42]. The extent of increase in the distances from the solvent above and below the cavity to the solute atoms will be larger for Cs^+^- than for Rb^+^-DB18C6 due to the higher position of Cs^+^ above the cavity. Those increases in the distances between the solute and solvent depending on the position of the metal cation above the cavity may lead to the decrease in the dispersion energy from K^+^- to Cs^+^-DB18C6.

## Theoretical methods

The initial conformations of M^+^-DB18C6 within the energy of 20 kJ/mol were searched using a Metropolis Monte Carlo method with the AMBER* force field in the Macromodel package (Schrödinger, LLC: New York) [7,12]. The geometries of those initial conformers were fully optimized by a series of theoretical calculations at the HF/3-21 G, HF/6-31 G, B3LYP/6-31 G, and then B3LYP/6-31 + G(d) levels. The optimization at the B3LYP/6-31 + G(d) level has been successfully employed to predict the lowest energy structures of DB18C6(H_2_O)_n_ (n = 0-4) [43], B18C6(H_2_O)_n_ (n = 0-1) [29], M^+^-DB18C6 [7], and M^+^-18C6(H_2_O)_n_ (M = Li, Na, K, Rb, and Cs, n = 0-4) [10,32,33]. The local minimum structures in aqueous solution were obtained by re-optimizing those optimized structures at the B3LYP/6-31 + G(d) level using the CPCM model and UAKS radii. The single point energy calculations at the B3LYP/6-311++G(d,p) level were then performed for all of the optimized structures at the B3LYP/6-31 + G(d) level. It was reported that the theoretical p*K*_a_ value obtained at the B3LYP/6-311++G(d,p)//B3LYP/6-31 + G(d) level using the CPCM agrees well with the experimental value of the Cys residue [44].

In all of those calculations, the Los Alamos effective-core potential LANL2DZ was used as a basis set for K, Rb, and Cs. No imaginary vibrational frequencies were found for all of the local minimum structures. The frequency calculations, zero-point energy (ZPE) corrections, and thermal energy corrections were performed at the B3LYP/6-31 + G(d) level with the scaling factor of 0.98 [45].

The BDFEs of M^+^-DB18C6 in the gas and solution phases were calculated for the following reaction:

(2) $$M^{+}\text{-}DB18C6\to M^{+}+DB18C6$$

The boat-shaped conformer of DB18C6 with C_2v_ symmetry was used in those calculations [29]. The counterpoise method was applied to correct the basis set superposition error [46]. All of the calculations were carried out using the Gaussian 03 suit [18].

## Conclusions

The BDFEs of M^+^-DB18C6 (M = Li, Na, K, Rb, and Cs) in aqueous solution are calculated using DFT and the CPCM model. The experimental binding selectivity of DB18C6 for alkali metal cations in aqueous solution is well reproduced by the theoretical BDFEs. It is found that the solvation energy of M^+^-DB18C6 plays a key role in determining the relative BDFE of M^+^-DB18C6 and hence the binding selectivity of DB18C6 in aqueous solution. Moreover, the non-electrostatic dispersion interactions between the solute and solvent, which strongly depend on the structure of M^+^-DB18C6, largely contribute to the different solvation energies of M^+^-DB18C6.

This study shows that the inexpensive continuum solvation model like the CPCM provides a tool to understand the binding selectivity of DB18C6 in aqueous solution on the basis of the followings: First, the strongest binding affinity of DB18C6 to K^+^ among other alkali metal cations as well as the decrease of the binding affinity with increasing or decreasing the size of the metal cation with respect to that of K^+^ are well reproduced by the calculations using DFT and the CPCM. Second, the solvation energy in the CPCM is given as a sum of electrostatic and non-electrostatic (cavitation, dispersion, and repulsion) energies, which is necessary to understand the relative importance of those interactions in determining the solvation energy. Third, the relative-energy order of the low energy conformers of M^+^-DB18C6, especially Rb^+^- and Cs^+^-DB18C6, in aqueous solution, which is obtained by geometry optimizations of the gas-phase conformers using the CPCM, turns out to be consistent with the experimental results [47]. More studies are under way to verify the feasibility of the CPCM model in predicting the binding selectivity of other host molecules in host-guest chemistry.

## Additional file

The following additional data are available with the online version of this paper. Additional data file contains the low energy structures of M^+^-DB18C6 in the gas phase calculated at the B3LYP/6-311++G(d,p)//B3LYP/6-31 + G(d) level, the Gibbs free energies of hydration of alkali metal cations predicted using the CPCM and MoCPCM models, and the BDFEs of M^+^-DB18C6 estimated using the MoCPCM.

## Competing interests

The authors declare that they have no competing interests.

## Authors’ contributions

CMC performed all of the calculations. NJK initiated and JH designed the study. JH and NJK analyzed the data and finalized the manuscript. All authors read and approved the final manuscript.

## Supplementary Material

Additional file 1 **Supporting Information Figure S1.The low energy structures of M**^**+**^**-DB18C6 in the gas phase.Table S1** Gibbs free energies of hydration for alkali metal cations in kcal/mol. **Table S2** Gibbs free energies for the dissociation of M^+^-DB18C6 in aqueous solution calculated using the MoCPCM model. (DOC 2545 kb) Click here for file
