# Supplementary material for: Binding selectivity of dibenzo-18-crown-6 for alkali metal cations in aqueous solution: A density functional theory study using a continuum solvation model
Source: Chem Cent J. 2012 Aug 8;6:84. doi: 10.1186/1752-153X-6-84 (PMC3464923; doi:10.1186/1752-153X-6-84)
Supplement: Additional file 1 — Supporting Information Figure S1.The low energy structures of M+-DB18C6 in the gas phase.Table S1 Gibbs free energies of hydration for alkali metal cations in kcal/mol. Table S2 Gibbs free energies for the dissociation of M+-DB18C6 in aqueous solution calculated using the MoCPCM model. (DOC 2545 kb) [file 1752-153X-6-84-S1.doc]

**Supporting Information**

**Binding Selectivity of Dibenzo-18-Crown-6 for Alkali Metal Cations in Aqueous Solution: A Density Functional Theory Study Using a Continuum Solvation Model**

Chang Min Choi^1^, Jiyoung Heo^2,*^, Nam Joon Kim^1,*^

^1^*Department of Chemistry, Chungbuk National University, Chungbuk 361-763, Korea*.

^2^*Department of Biomedical Technology, Sangmyung University, Chungnam 330-720, Korea*

**Fig. 1S.** The low energy structures of M^+^-DB18C6 in the gas phase


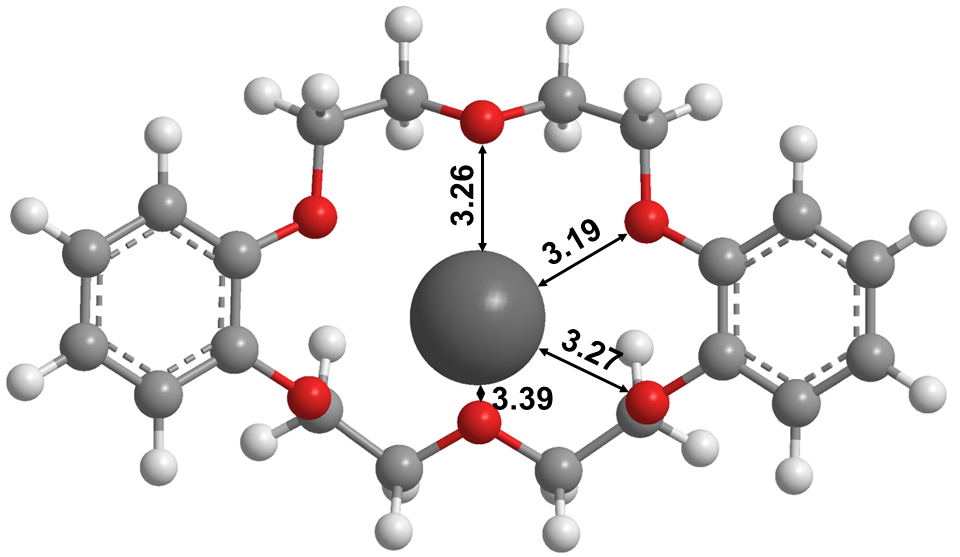

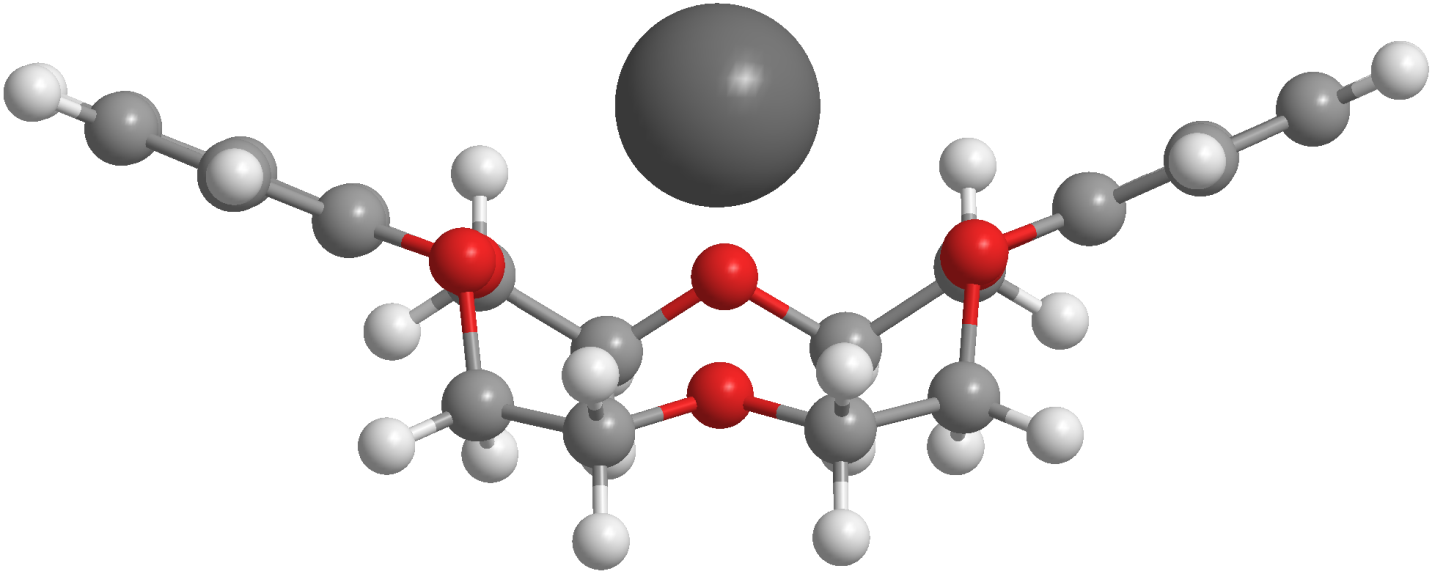


Cs-I


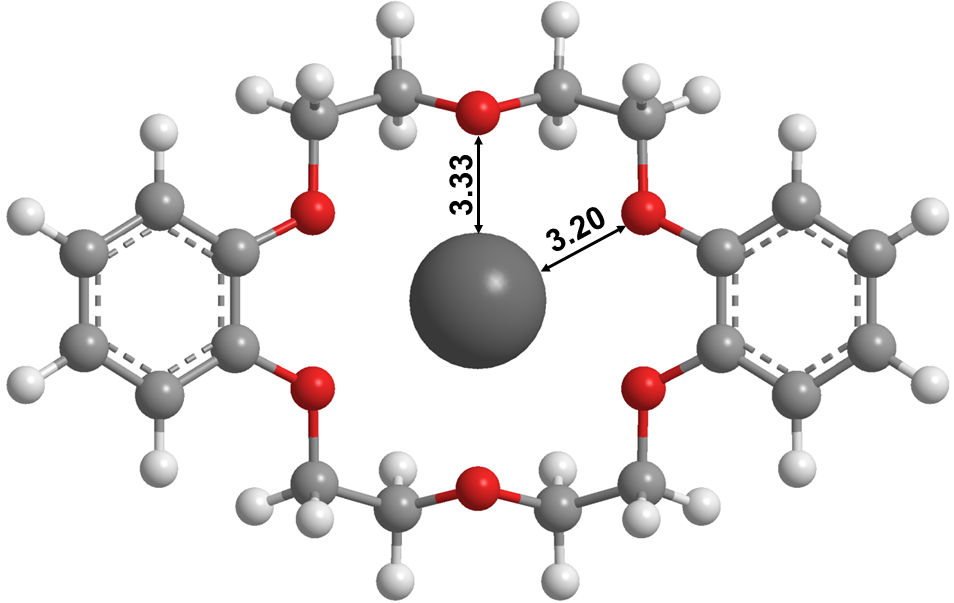

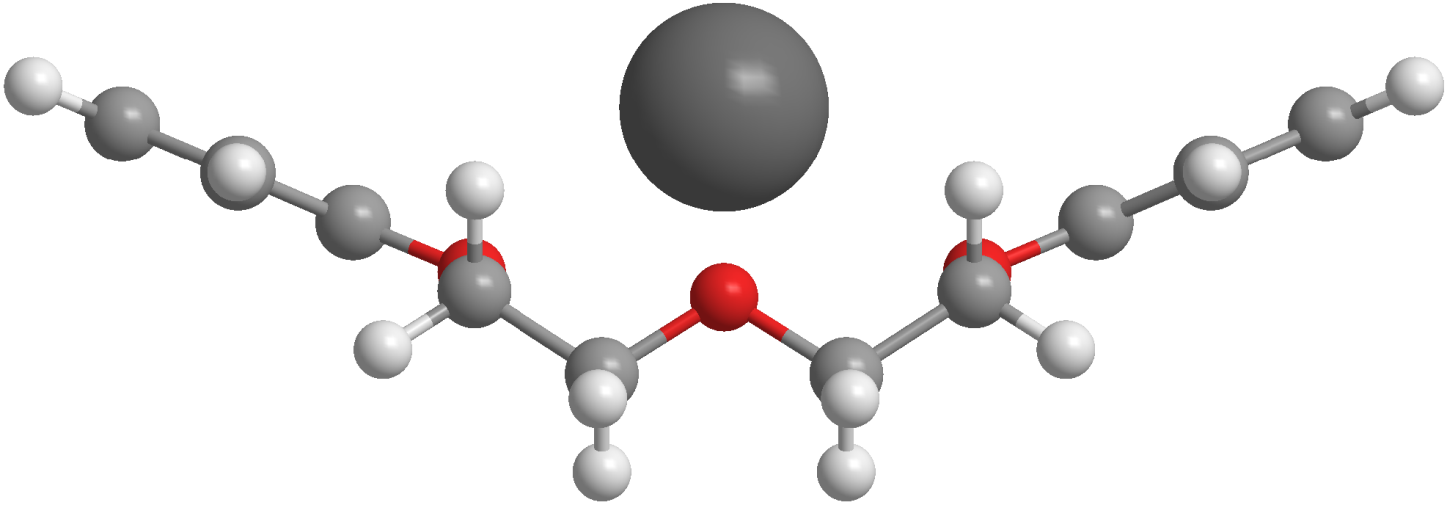


Cs-II


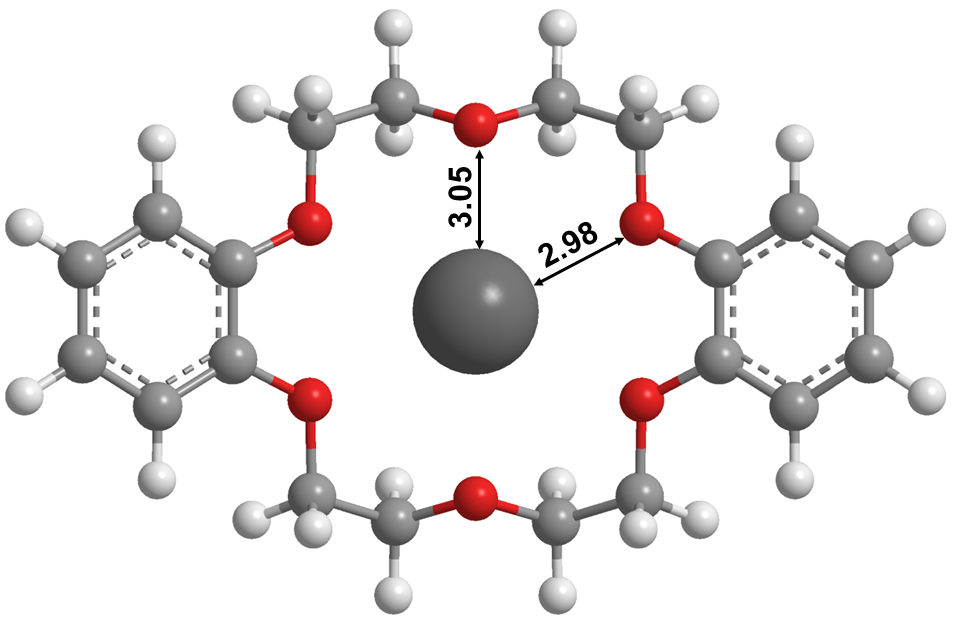

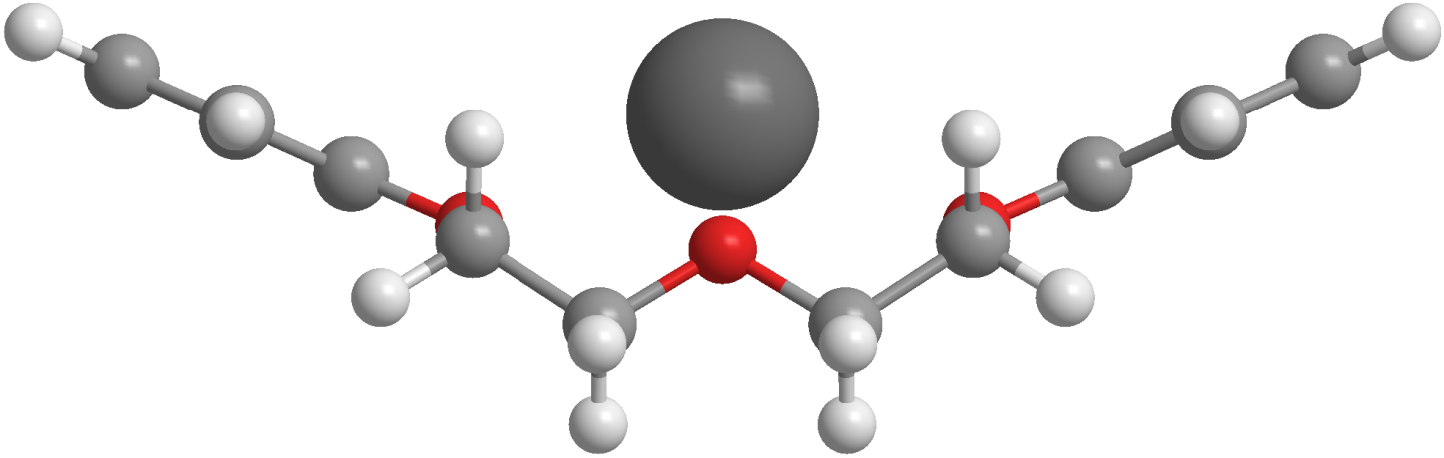


Rb-I


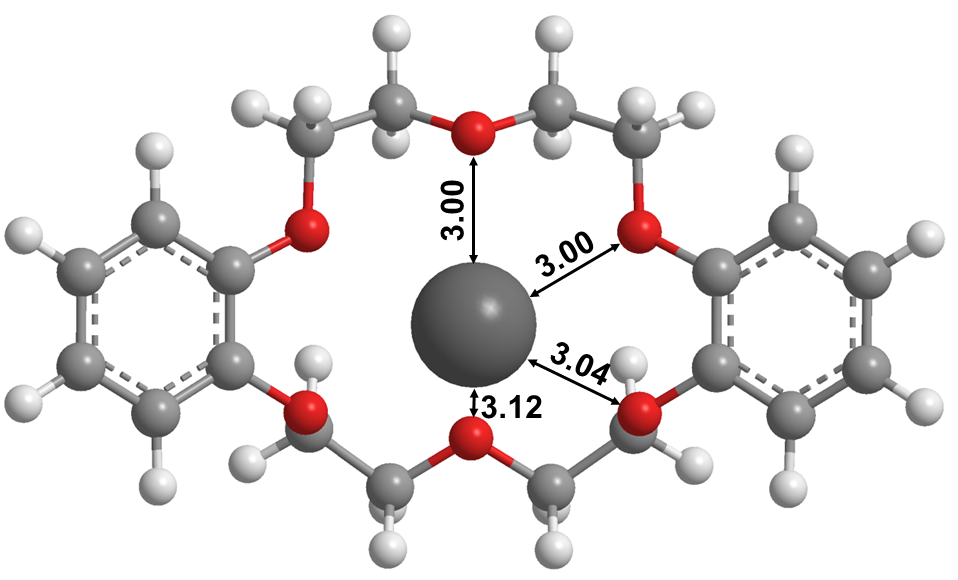

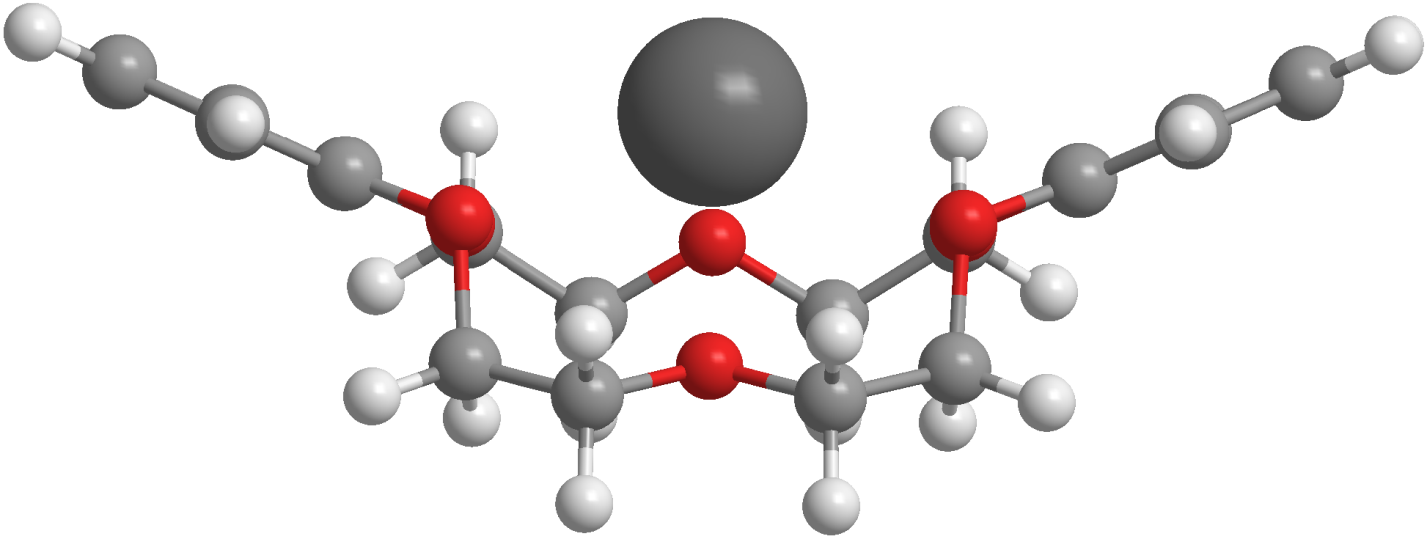


Rb-II


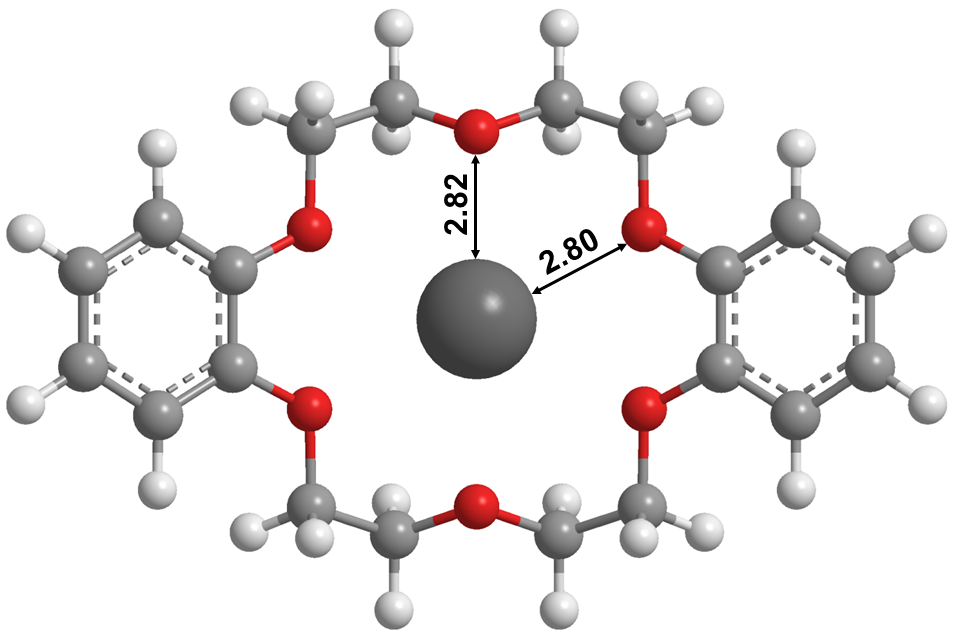

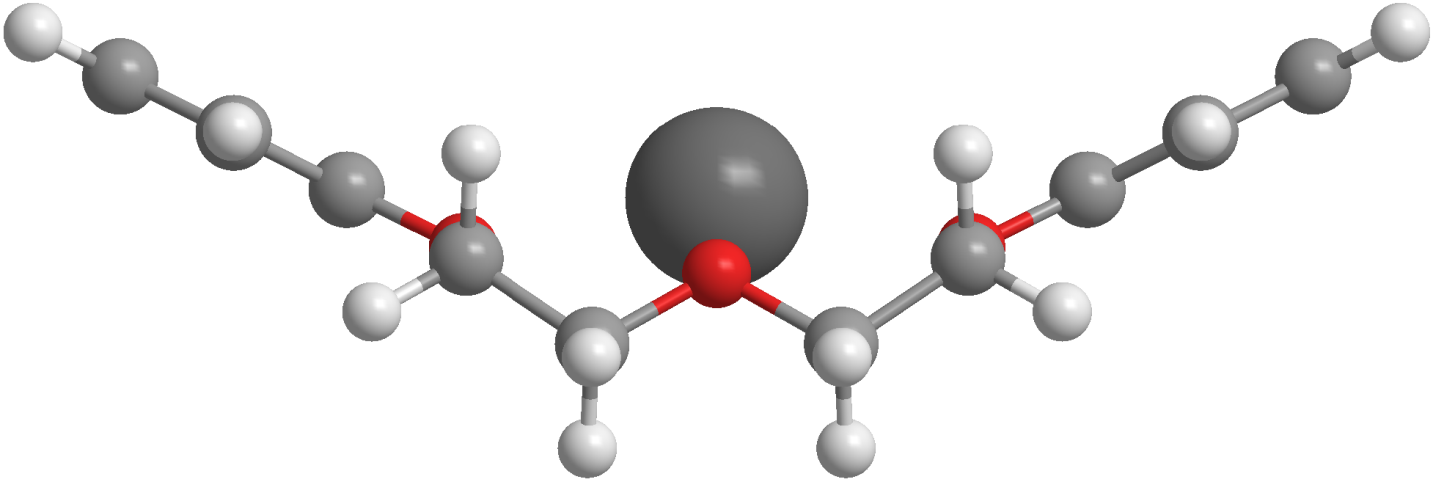


K-I


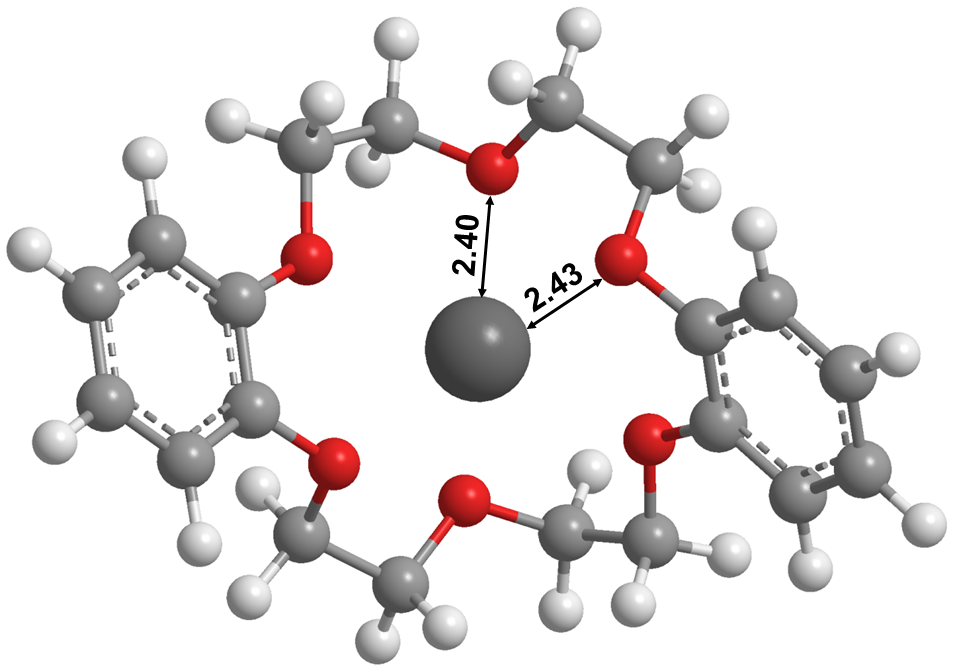

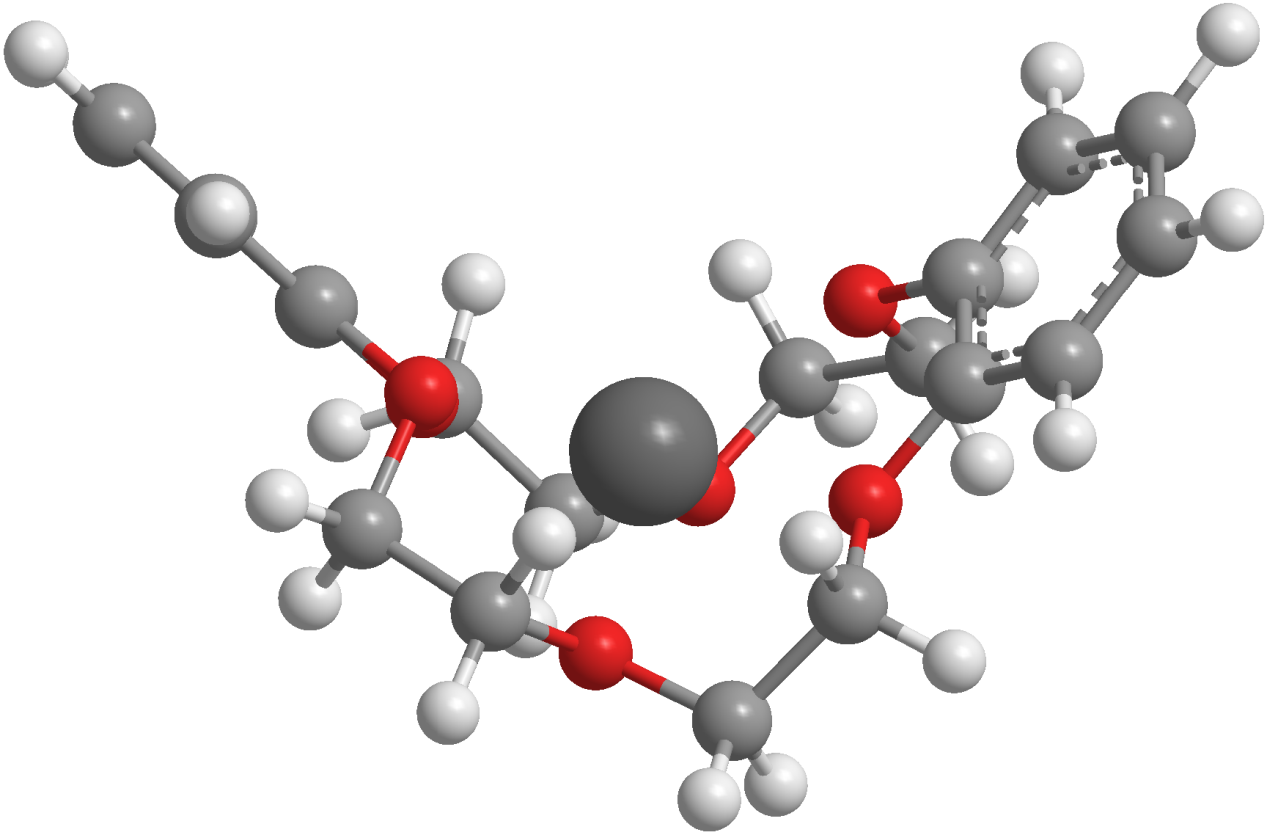


Na-I


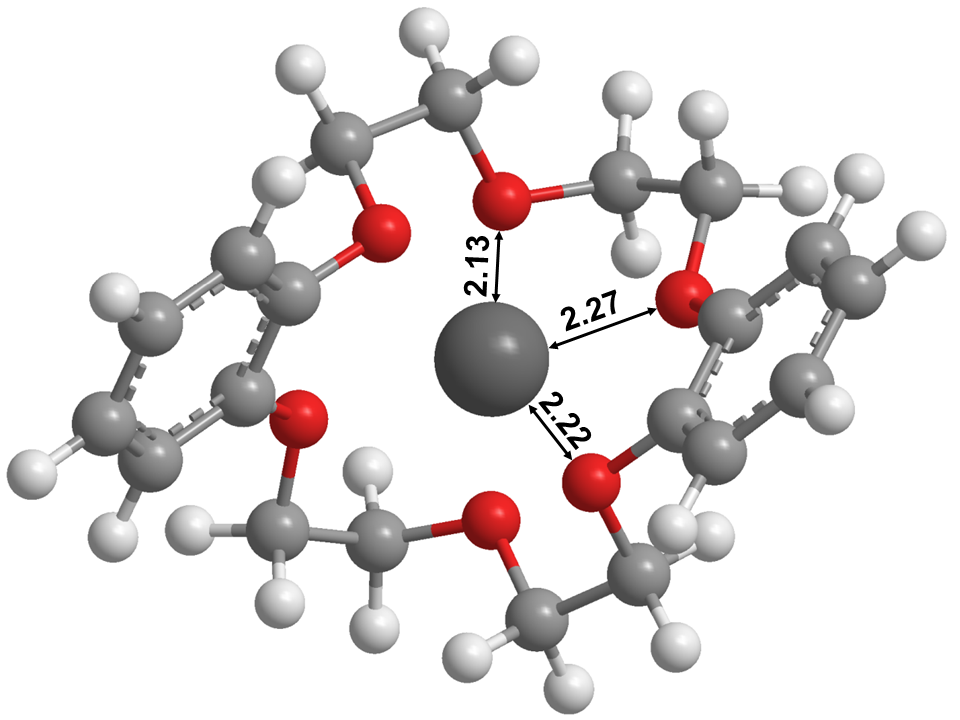

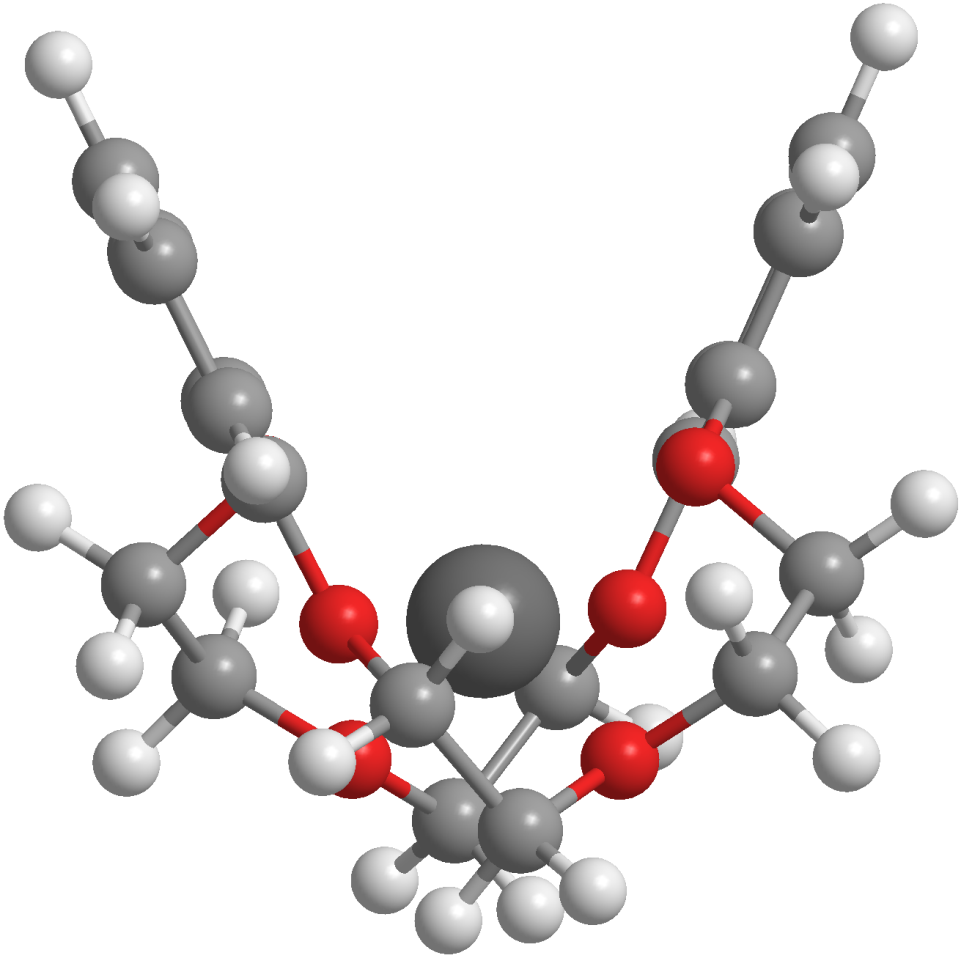


Li-I

**Table S1**

Gibbs free energies of hydration for alkali metal cations in kcal/mol

|  | CPCM | |  | MoCPCM | |  | Exp*^a^* |
| --- | --- | --- | --- | --- | --- | --- | --- |
|  | *r^b^* | Δ*G*_s_ |  | *r* | Δ*G*_s_ |  | Δ*G*_s_ |
| Li^+^ | 1.23 | -108.66 |  | 1.10 | -121.79 |  | -122.10 |
| Na^+^ | 1.49 | -89.35 |  | 1.37 | -98.09 |  | -98.20 |
| K^+^ | 1.91 | -69.98 |  | 1.69 | -80.76 |  | -80.60 |
| Rb^+^ | 2.06 | -60.28 |  | 1.72 | -75.43 |  | -75.50 |
| Cs^+^ | 2.26 | -53.34 |  | 1.89 | -67.87 |  | -67.80 |

*^a^*Ref. 1. *^b^*Sphere radii of alkali metal cations.

**Table S2**

Gibbs free energies for the dissociation of M^+^-DB18C6 in aqueous solution calculated using the MoCPCM model*^a^*

|  |  | Δ*G* |
| --- | --- | --- |
| Li | I | -10.38 |
|  | II | -10.91 |
| Na | I | -5.25 |
|  | IV | -4.79 |
|  | V | -4.98 |
| K | I | -4.02 |
| Rb | I | -9.08 |
| Cs | II | -11.62 |

*^a^*Units in kcal/mol.

**References**

1. Burgess MA: *Metal Ions in Solution* Chichester, England: Ellis Horwood; 1978.
